# Qualitative assessments of anemia‐related programs in Ghana reveal gaps and implementation challenges

**DOI:** 10.1111/nyas.14538

**Published:** 2020-12-24

**Authors:** Brenda A.Z. Abu, Nicole Buttner, Olivia D. Garror, Rachel Stefanic, Adam Sandow, Kinglsey A. Pereko

**Affiliations:** ^1^ Rochester Institute of Technology, College of Health Sciences and Technology Wegmans School of Health and Nutrition Rochester New York; ^2^ Point Hope International Ghana Program Kasoa Ghana; ^3^ Community Medicine University of Cape Coast School of Medical Sciences Cape Coast Ghana

**Keywords:** conceptual framework of malnutrition, multisectoral approach, anemia, children, programs/projects

## Abstract

In spite of multiple program efforts in Ghana, progress in reducing the burden of anemia is slow. The objective was to conduct multilevel assessments of existing childhood (<5 years) anemia prevention and treatment programs according to UNICEF's conceptual framework of malnutrition, and to elucidate implementation gaps in Ghana. Purposive and snowball sampling strategies recruited 25 program personnel from 20 organizations to participate in audiorecorded interviews conducted through in‐person, telephone, or email correspondence in August 2018. Interview guides constructed around UNICEF's conceptual framework of malnutrition identified context‐specific immediate, underlying, and basic causes of anemia, and corresponding programs. Interviews were transcribed, coded, and analyzed using the Dedoose software version 8.1.8. Few programs addressed identified basic causes of anemia, such as inadequate human resources, housing/water/toilet facilities, and poverty/poor access to financial resources. Organizations implemented programs addressing ≥1 underlying cause. Five organizations provided food rations and/or supplements to address immediate causes. A key food‐based gap identified was minimal education on fruit intake or antinutritive factors in foods; however, no interventions included vitamin C supplements. Food manufacturers mainly used cereals and grains in commercial food products. Multiple organizations worked in the same region on anemia with instances of an overlapping program focus. Food sources of vitamin C or supplements could be promoted in food‐based interventions to increase the absorption of nonheme iron consumed.

## Graphical abstract

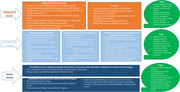

## Introduction

Globally, anemia is a leading cause of morbidity and mortality among women and children. In 2011, 43% of children under 5 years, 38% of pregnant women, and 29% of nonpregnant women between ages of 15 and 49 years were living with anemia. In South Asia, and central and west Africa, program efforts have reduced anemia rates to a lesser degree than expected.^1^ The consequences of anemia include delayed mental development, lost wages related to illness and lethargy, increased healthcare cost, and impaired economic development. According to UNICEF's conceptual framework, the causes of malnutrition are multileveled and can be categorized as immediate, underlying, and basic.^2^ To address anemia holistically, sufficient program efforts must address contextual causes at all three levels.

Anemia affects 66% of children (6–59 months) in Ghana, according to the National Survey in 2015. Iron deficiency (ID) resulting from inadequate dietary iron intake and infections are leading causes of nutritional anemia in Ghana.^3^ Current data (2018) indicate that 35.6% of preschool children had anemia, 21.5% were iron‐deficient, and 12.2% had ID anemia.^4^ In Ghana, malaria affects 26.7% of children and helminthic infections because of parasitic worms that cause 45% of anemia cases.^5^ Helminth and malaria infections cause malabsorption of nutrients, inflammation, blood loss, and reduced appetite, all of which exacerbate anemia status. Other nutrient deficiencies, such as a deficiency in vitamin B_12_, folate, and vitamin A, usually concurrently exist in patients with anemia.^5^, ^6^, ^7^ Maternal anemia is a risk factor for anemia in children 6‐ to 59‐month olds^1^ in Malawi, Mozambique, Namibia, Zimbabwe,^8^ and Ghana.^3^

Nutrition‐specific and ‐sensitive interventions are used to address anemia in the global context.^9^ In parts of Asia and Africa, agricultural interventions, such as home gardens^10^ and irrigation, improved food security and anemia rates.^11^ Water Sanitation and Hygiene (WASH) interventions implemented in schools and homes have also been shown to reduce the rates of anemia.^12^, ^13^ Some nutrition‐specific interventions include distribution of micronutrient powders (MNPs),^14^ milk fortification,^15^ and supplementary food assistance to vulnerable women, infants, and children.^16^ Ready‐to‐use therapeutic foods (RUTFs) and complementary feeding programs have also been used in the prevention and treatment of malnutrition and anemia.^17^ In Ghana, programs, such as the USAID Strengthening Partnerships, Results and Innovations in Nutrition Globally (SPRING) project, focused on multiple interventions, such as nutrition, reproductive health, disease control, agriculture, WASH, and education to address anemia.^18^, ^19^

In Ghana, numerous preventive and management interventions are implemented concurrently to address childhood anemia. Despite existing program efforts, the incidence of childhood anemia remains high. The objectives of our study were to conduct multilevel assessments of existing childhood anemia prevention and treatment programs, to elucidate implementation gaps in Ghana using UNICEF's conceptual framework of malnutrition as a guide.

## Materials and methods

### Study design and sampling

In our cross‐sectional qualitative study, purposive sampling techniques were used to identify organizations currently implementing childhood anemia prevention and treatment programs. These organizations were identified from earlier studies conducted by the researchers, and desktop review. Snowball sampling procedures were used to identify additional stakeholders.

Stakeholders were identified from national, regional, district, and subdistrict levels using the above recruitment methods. Organizations were contacted and invited to the study. Interested organizations then chose one personnel to represent their program. In some cases, project teams represented one organization. The selected representatives of the organizations were the technical expert, program officer, and/or project manager responsible for a nutrition and/or anemia‐related project. Interviewed representative(s) identified other relevant organizations that they worked with or knew to work in anemia‐related programs. The listed stakeholder was also then contacted and invited to the study. Interested organizations responded with a confirmation on their willingness to participate and possible available times for an interview. This snowball sampling technique increased the diversity of the sample. Recruited stakeholders included health service providers, hospitals, refugee camp management, university faculty, individuals, and organizations involved in food fortification and processing, and national regulatory organizations.

### Ethical considerations

This project protocol was reviewed and approved by the Institutional Review Board (IRB) of the Rochester Institute of Technology (FWA# 00000731) and the Ethical Review Committee of the Ghana Health Service (GHS; GHS‐ERC003/07/18). Only organizations that responded, consented, or gave permission for audio recordings of discussions were interviewed. A data collection method (in‐person, phone, and email) was self‐selected based on the availability of program personnel, thus, allowing the opportunity to interview more programs. An incentive in the form of a gift card was offered to participants who responded to the survey outside of their office hours.

### Inclusion and exclusion criteria

All organizations implementing treatment or prevention of anemia‐related programs in Ghana were eligible. Service providers, public health organizations, hospitals, regulatory officers, and food manufacturers were selected by their organizations to participate in the study.

Exclusion criteria were defined as organizations that did not implement any health, nutrition, or anemia‐related interventions to address one or more of the causes of childhood anemia in Ghana, according to UNICEF's conceptual framework.^2^ Hospitals/medical facilities that provided only emergency care or did not treat anemia‐related conditions were excluded.

### Data collection tools and techniques

Program profiles, target population, strategies, and coverage were elicited using interview guides. Interview guides were developed using tenets from UNICEF's conceptual framework of malnutrition.^2^ Additional open‐ended questions such as “What are the challenges you encounter during the implementation of anemia related interventions?” and “What do you think are the program gaps in childhood anemia interventions?” were posed to respondents to collect information on the perceived implementation challenges and gaps in services. Interview guides were created for each of the following four groups: (1) program and project implementing agencies; (2) medical facilities and biomedical laboratories; (3) food processors and manufactures; and (4) regulatory agencies. The interview guides were pilot tested and assessed for face and content validity before they were used for interviews. Three research assistants (N.B., O.G., and R.S.) and the lead researcher (B.A.Z.A.) conducted data collection. Copies of the four interview guides are attached as Supplementary Material (online only).

During data collection, the corresponding interview guide was shared with program personnel before or during the interview. The personnel/organization used the interview guide to prepare in advance for the interview. Program resources were made ready for review during interviews. Interviews were conducted in‐person, over the phone, and through surveys submitted via email correspondences in August 2018. A selected data collection technique was used based on the availability of program staff. For email responses, the interview guide was shared with the program officer who provided in‐depth written responses to the questions. In‐person and over‐the‐phone interviews were recorded using Olympus DS‐2500 digital voice recorders and hand‐written notes. Each interview lasted for about 1 hour. Participants were recruited from universities, local nongovernmental organizations (NGOs), government departments, food manufacturing companies, and international agencies. Data were collected in the Greater Accra, Cape Coast, and Northern regions of Ghana.

During interviews, observations and field notes were made to validate context‐specific issues, such as food preparation techniques, safety and hygiene, vending and restaurants in communities, advertisement and marketing, and food labels. These observations were used to make deductions during assessments of the programs in discussing the findings of this study.

### Data analysis

A professional transcriber transcribed the audiorecorded interviews. Four of the researchers (B.A.Z.A., N.B., O.G., and R.S.) coded the content of the transcriptions using the Dedoose software version 8.1.8. The coding team conducted the initial review of all transcripts and created themes according to the questions in the interview guides. The themes were incorporated into the Dedoose coding system. Responses from transcripts were categorized into respective themes (major codes). Responses of subthemes (child codes) were used to further group codes. Excerpts (quotations) were highlighted to match each theme and subtheme. A systematic process was used to discuss and synchronize themes, code names, and subthemes during a series of meetings. Key themes and codes were reported in the results.

## Results

### Description of the organizations, programs, and respondents

Twenty‐five respondents from 20 organizations, including universities (*n = *2), local NGOs (*n = *4), government departments (*n = *6), local hospitals/clinics (*n* = 3), food manufactures (*n = *2), and international (*n = *3) agencies, completed interviews as indicated in Table 1.

**Table 1:** Participating organizations and their contributions to childhood anemia

Organizations	Key projects/contribution to childhood anemia prevention, management, and treatment	Scope of implementation
Universities (*n* = 2)		
*^a,^*, *^b^*University of Cape Coast	Grant‐funded research	National
University of Ghana	Training of health personnel	
	Community outreach programs	
Governmental departments (*n* = 6)		
*^a,^*, *^b^*The GHS, Accra (a team of three)	General nutrition education	National
*^a^*The GHS, Cape Coast (a team of two)	Draft standards and regulations of food processors product labeling (infant formula, cereals, snacks, and supplements)	
The GHS, Ampain	Draft standards and regulations on housing	
The FDA, Accra	Regulation on water and sanitation	
The GSA, Accra	Policing compliance of food vendors and grocery shops on food labeling and safety regulations	
*^a^*The GSFP, Accra (a team of four)	Provide school meals to 1/3 of the RDAs of elementary school children	
	Pilot the homegrown school feeding program in 10 schools in each region Provide folic acid and iron supplements to women and vitamin A and iron supplements to children	
Local hospitals/clinics (*n* = 3)	Provide ready‐to‐use‐therapeutic food (RUTF) supplement for moderately malnourished children Provide a therapeutic diet (F‐75 and F‐100) for in‐patients with anemia and protein energy malnutrition (PEM) Provide dietary supplements (BP 100 or Plumpy'Nut) provided to children with severe malnutrition Provide iron and iron‐related vitamin supplementation Provide nutrition education to caregivers of admitted children Provide blood transfusions for severe anemia cases	In‐ and out‐patients in the Cape Coast area
*^a^*Cape Coast Regional hospital (a team of three)		
New Ebu subdistrict hospital		
Food manufacturers (*n = *2)		
Sight and Life Foundation	Produce commercialized fortified food products for the *Obaasima* project that fortifies cereal, biscuit, and hot sauce (*shito*)	National (headquarter is based in Accra)
Finer Food Company Ltd.	Processed cereal porridges	Pilot in Northern and Brong Ahafo regions in Ghana
Local nongovernmental (*n* = 4)		
*^a^*New Ebu Child Development Center (a team of three)	Provide Water, Hygiene, and Sanitation (WASH) education to young children	Cape Coast Area,
The Christian Association of Ghana *^b^*Point Hope Ghana, Buduburam *^b^*Point Hope Ghana, Krisan	Provide general nutrition education to refugees Maintain a community gardening project	Refugee populations in Buduburam, Krisan, and Ampain
International agencies (*n* = 6)		
The RING–USAID (a team of five)	Fund community‐based organizations implementing nutrition projects Provide technical expertise on anemia assessment Provide communities with WASH projects Provide communities with gardening projects	National
The USAID (a team of three)	Provide laboratory equipment and train health personnel for assessment of anemia Support Village Saving and Loans Associations (VSLA)	Northern Ghana
The GRB (UNHCR) Ampain Krisan Buduburam	Facilitate access to housing and water Provide safe water at subsidized prices for the refugee population through water users associations	Refugees in Ampain, Krisan, and Buduburam

^*a*^ More than one respondent was present during one interview.

^*b*^ Multiple project personnel were interviewed from one organization.

GHS, Ghana Health Service; GSFP, Ghana School Feeding Program; GRB, Ghana Refugee Board; *Obaasima*, “a virtuous woman” in the Akan language; RDA, recommended dietary allowance; RING, Resiliency in North Ghana; USAID, the United States Agency for International Development.

University lecturers contributed to the prevention of childhood anemia through research, training of health personnel, and outreach activities. All three of the faculty members that were interviewed were conducting research addressing various levels of childhood anemia. Some of these research projects were targeted at the maternal health of fishmongers, improving food security using poultry and gardening, and financial literacy for adolescent girls. Universities provided academic and skills training of medical officers, public health officers, nutritionists, and dietitians. Three forms of training modalities that influenced anemia treatment were reported as academic content for the future health professionals, student research projects, and experiential training and outreach. Students were recruited for these health programs countrywide.

Governmental departments interviewed served as the national overseers and provided services for the health needs of the country. The GHS provided nutrition education to women, and community‐based growth monitoring and promotion of children under 5 years. The Ghana School Feeding Program (GSFP) provided one hot meal per day to meet a third of the recommended dietary allowance of nutrients and calories for children. Additionally, the GSFP provided safe drinking water for the beneficiary elementary schools and as part of the program. The GSFP piloted a homegrown school feeding program to promote the use of locally grown food crops. The Ghana Standards Authority (GSA) responsible for drafting standards used by food manufactures when creating product labels of infant formula, cereals, and snacks equally contributed to prevention efforts. The GSA also drafted standards for housing that mandated that each house must be constructed with toilet facilities and access to public water. The Food and Drugs Authority (FDA) regulated the standards set by the GSA regarding food safety, food processing, labeling, and medication and supplements, such as iron, vitamin A, vitamin C, and folate. Most of these processed foods were made from plant staples. The GHS Nutrition Officer in a refugee settlement confirmed the common use of plant staples in food products in her statement:

“Sometimes we have something [food] called seri‐soya that is mixed components of maize, beans, rice, yeah, millet, yeah, blended together, they use that as porridge for children and it's very nutritious.”

The community clinics and hospitals mainly provided treatment to children who were diagnosed with anemia and protein energy malnutrition (PEM). Standard PEM management methods include medication, supplements, therapeutic diets, and blood transfusion in severe anemia cases. Therapeutic feeding regimens include F‐100 and F‐75 milk‐based nutritional supplements for inpatients. Other clinics used RUTFs that were high‐energy micronutrient (MN)‐enriched pastes with a similar nutrition profile as the F‐100 milk‐based nutritional supplements. These ready‐to‐use therapeutic food (RUTFs) were provided to children at discharge from hospital admissions, and to outpatients during follow‐up treatment visits. Nutrition education on healthy eating was also provided to caregivers.

Food processors mainly contributed to addressing childhood anemia through food processing and sale of iron‐rich foods, and complying with the mandatory or voluntary fortification of food products guidelines. The Sight and Life Foundation collaborated with the German Development Cooperation (GIZ), the Association of Ghana Industries, and the GSA to produce fortified ready‐to‐eat milk biscuit; fortified ready‐to‐eat vegetable sauce; fortified ready‐to‐eat corn‐soy blend. The collaborative project, also called “*Obaasima*” (meaning “a virtuous woman” in the Akan language), had a goal of providing Affordable Nutritious Foods For Women (ANF4W). The small‐scale local processor interviewed mainly vended cereal powders in different formulations using rice, maize, beans, sorghum, millet, and soybeans.

Additionally, one of the local NGOs interviewed was a church‐based institution that provided spiritual, financial, and material support to sponsored children in rural communities. About 400 children were enrolled in this program. Another NGO provided nutrition services and preschool facilities in the refugee camps and settlements in Buduburam, Ampain, and Krisan. The NGO in the refugee setting maintained a community garden, fish pond, and livestock used to improve the nutrition of preschoolers and at‐risk households. At‐risk households were mainly households with a child who was admitted and/or treated for severe malnutrition.

The U.S. Agency for International Development (USAID) provided funding for WASH and nutrition‐related projects. One of such projects is the Resiliency in Northern Ghana (RING) project that provided technical expertise to assemblies and health personnel in Northern Ghana that implemented projects on nutrition education, irrigation, and gardening. The RING project provided hemocues and test kits for anemia diagnosis as part of their on‐the‐job training.

The Ghana Refugee Board, through support from the United Nations Refugee Commission (UNHCR), enables access to housing, water, and vending space for refugees. The GHS provides onsite health and treatment for women and children in the camp. The refugee camp daycare Program officer said:

“Since it's a refugee camp, we keep the children so that the parents would be able to go out and look for money and buy food for dinner. So, we give them [children] breakfast and lunch every day.”

Table 2 summarizes the nutrition training background of personnel involved with program development and implementation. Out of the 25 personnel interviewed, only six of them had not previously received some academic or professional training on nutrition and anemia prevention. Their key roles, however, demanded expertise or at least sensitization on anemia‐related issues.

**Table 2:** An inventory of education, training, and responsibilities of personnel in childhood anemia–related programs in Ghana

Name of organization	Key respondent's position	Highest education	Anemia‐related training	Role in program design/development/implementation/monitoring
**Implementing agencies and projects**	*n* = 20			
University of Cape Coast School of Medical Sciences	Senior lecturer in nutrition sciences	PhD in Nutrition	Yes	Teach nutrition, training students, and conduct research on environmental health issues
The School of Public Health, University of Ghana	Senior lecturer in nutrition sciences	PhD in Human Nutrition	Yes	Teach nutrition, training students, and conduct research on maternal and child health
University of Cape Coast School of Medical Sciences	Head of Clinical Nutrition and Dietetics Department	PhD in Population and Health	Yes	Teach nutrition, training students, and conduct research on anemia, and food security
GHS/Ampain	Midwife	Certificate in Midwifery	Yes	Provide prenatal and postnatal nutrition education
GHS–Ampain	Midwife	Certificate in Midwifery	Yes	Prenatal and postnatal anemia assessments, testing, and counseling
GHS–Cape Coast	Community health officer	Diploma in community health	Yes	Prenatal and postnatal anemia assessments, testing, and counseling
GHS–Accra	Regional nutrition officer	MPhil in nutrition	Yes	Nutrition program development, monitoring, and evaluation
Buduburum refugee camp	Housing and utility manager	HND	No	In‐charge of refugee camp housing, utility, and permits for vending space
Buduburum refugee camp	Water vendors	HSC	No	Coordinate subsidized water sales
GRB/Ampain	Nutrition officer	MS in food and nutrition	Yes	Provide nutrition, education, and counseling
GRB/Ampain	Camp manager	HND	No	In‐charge of refugee camp housing, utility, and permits for vending access
GRB/Krisan	Camp manager	Diploma in marketing	No	In‐charge of refugee camp housing, utility, and permits for vending access
GHS−Cape Coast Regional Office	Nutrition officer	BEd in home economics	Yes	Nutrition program development, monitoring, and evaluation
GSFP	Head, GSFP	MD	Yes	Develop strategies to implement the GSFP
Point Hope Ghana−Krisan Daycare	Project officer	BS in home science	Yes	Provide a hot meal a day to children
Point Hope Ghana	Beneficiary coordinator	BS in home science and psychology	Yes	Nutrition, education, health, and skills training
Christian Association of Ghana	Nutrition officer	Diploma in public health nursing	Yes	Provide general nutrition education
RING−USAID	Chief of party	MS in environmental sciences	Yes	The coordinate RING project focused on women in their reproductive age and children ≤5 years. The goal of reducing stunting, wasting, and anemia, underweight among children under age 5
New Ebu Child Development Center	Center director	Postgraduate diploma−wireless mobile communication	No	Provide holistic child development services: spiritual, community, social, emotional, and physical development (including nutritional status) of the child
USAID	Head of Nutrition Department	MS in nutrition	Yes	Provide grants and technical expertise on nutrition projects
**Regulatory agencies**	*n* = 2			
GSA	National enquiry point officer	MS in development management	No	Coordinated expert meetings to developed standards across the board
FDA	Senior regulatory officer	MPhil in nutrition	Yes	Enforces mandatory fortification of wheat flour with micronutrients (iron, folic acid, vitamin A and vitamin B, and zinc), and vegetable oil with VA as a preventive measure to address iron and VA deficiency in Ghana
**Medical facilities and biomedical laboratory**	*n = *1			
Cape Coast Teaching Hospital	Dietitian	MS in nutrition and dietetics	Yes	Provide clinical nutrition treatment to children
**Food producers and manufacturers**	*n = *2			
Sight and Life Foundation	Food fortification expert	MPhil in food science	Yes	Facilitate partnerships with, Development Corporation (GIZ) and Bill & Melinda Gates Foundation, Association of Ghanaian Industries (AGI), and GSA to implement the OBAASIMA project
Finer Foods Company Ltd.	Director	MS dietetics	Yes	Cereal product formulation, processing, and labeling

BEd, bachelor of education; BS, bachelor of science; FDA, Food and Drugs Authority; GHS, Ghana Health Service; GRB, Ghana Refugee Board; GSA, Ghana Standards Authority; GSFP, Ghana School Feeding Program; HND, higher national diploma; HSC, high school certificated; MS, master of science; MPhil, master of philosophy; PhD, doctor of philosophy; RING, Resiliency in Northern Ghana; USAID, the U.S. Agency for International Development.

### Assessment of implemented childhood anemia programs according to the conceptual framework of malnutrition

**Figure 1:**
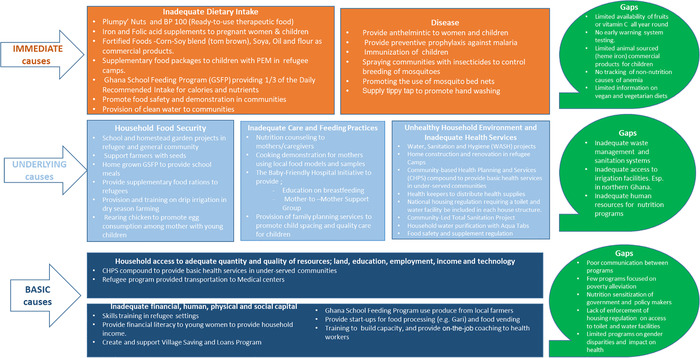
Projects/programs addressing the basic, underlying, and immediate causes of anemia in Ghana, and identified gaps. Adapted and modified from UNICEF's conceptual framework of malnutrition.^2^ Note that no connections exist between programs, unlike the connections indicated in the framework.

The programs implemented have been grouped using UNICEF's conceptual framework of malnutrition (Fig. 1), indicating the three levels of the causes of malnutrition (anemia): basic, underlying, and immediate causes.

Immediate causes of childhood anemia unique to Ghana were malaria, worm infestation, diarrhea, and inadequate dietary quality. All organizations implemented programs addressing one or more of the immediate causes. Many overlapping programs from different organizations addressed inadequate dietary intake in the same district. A USAID RING program officer puts this in perspective:

“Sometimes depending on the beneficiaries [the demand] is too great, we work in USAID, I go with my WASH project, they come with their nutrition to the same person, another person come with their Agric or education project, what time would the beneficiary get to attend to the farm and even now attend to the child.”

Programs included provisions of subsidized water, food through school feeding, iron and folate supplements to pregnant women, and sale of commercially fortified foods. The RING project reported that they provided anthelmintics according to the WHO guidelines. The GHS and RING project provided complementary public health assistance to prevent anemia through VA supplementation, immunization, and promotion of the use of bed nets.

Underlying causes of childhood anemia unique to Ghana were food insecurity, inadequate access to health services, especially in the rural settings, low‐quality food safety and environmental hygiene, food insecurity, and inadequate nutrition and care knowledge, especially in the rural farming and fishing communities. Some of the food security–related interventions were WASH projects, community and household gardening, and the provision of supplementary foods to refugee populations. Government‐assisted programs and universities reported program reached national scope; nongovernmental and private organizations implemented regional/district focus interventions that addressed these contextual factors because of limited funding.

All five of the basic causes of childhood anemia listed in UNICEF's conceptual framework were reported to affect anemia prevention and treatment in Ghana, indirectly. These causes included impaired household access to adequate and quality resources, such as education, housing, employment, skilled health personnel, health facilities, and sociocultural factors. Gender roles and cultural practices contributed to the manner in which household food was shared, consequently, men of the households were given larger and better portions of meat than women and children. The practice was reported as being accepted among most ethnic groups in Ghana. Thus, indirectly influencing the intake of heme iron by women and young children. Organizations that provided supplements also provided education to address misconceptions regarding the use of these services, as well as cultural practices. Although Ghana had a stable economy, the respondents believed that governmental policy commitment did not prioritize nutrition issues. For instance, many rural communities lacked access to a good road and storage facilities important for consistent access to food, markets, and nonfood essentials. Additionally, most communities relied on the Community‐based Health Planning and Services (CHPS) compound, supported by the GHS, NGOs, and the community members for their health needs. Community health nurses trained to provide first aid for ailments mainly independently managed the CHPS compounds, rendering most rural communities with limited access to advanced care.

### Perceived program implementation challenges and gaps as reported by participants

Reported challenges in anemia prevention included insufficient funding for implementation of projects, sociocultural practices, and beliefs, the seasonality of crops, and endemic poverty, especially in rural areas. Many respondents indicated that a major challenge in anemia prevention was inadequate funding for projects. Many organizations shared ideas and strategies for preventing anemia on a large scale; however, they believed that financial support from the government and the private sector was inadequate to implement large‐scale sustainable projects.

The health personnel perceived that current sociocultural practices also contributed to the challenges of anemia prevention and treatment. Some local traditions and cultures rejected certain nutrient‐dense foods, for example, denying children meat, for fear that the consumption of meat will cause the children to become thieves. In a few communities, people yet did not believe in seeking orthodox medicine when ill, and instead, they used traditional herbs. The respondents believed that these health‐seeking behaviors reduced the impact of program strategies.

According to the respondents, program implementation in rural areas, especially in the Northern and Central regions of Ghana, was challenging because of the overwhelming poverty in larger proportions of the households. They reported that although their organizations provided education to the public regarding how to improve nutritional status and diet, many lacked the financial ability to afford diverse nutrient‐rich meals. Program officers at the community level reported overall high percentages of households with financial constraints limiting access to iron‐rich foods, especially in rural communities. An official from the USAID−RING project put this assertion in perspective:

“…I think so far, we can't just point out one gap because the issues are many and we all know anemia is a general health situation. I think from what we have shared so far, we will say poverty goes to cause lack of education, and lack of services is a key gap…”

The health personnel believed that providing education was often not tailored to each community context, such as using available and affordable food sources in each community as examples, making it difficult for individuals to comply. The dry season in the Northern sector of Ghana lasted for half a year. During this dry season, many fresh crops, vegetables, and fruits were unavailable. This natural phenomenon contributed to the persistent food insecurity in rural communities in the area.

Inadequate health personnel to provide health services was reported as another challenge. In addition to this, the NGO that provided on‐the‐job training for clinical health personnel reported that trained staff were frequently transferred to new locations, interrupting the continuity of care. Program personnel at the regional hospitals reported that doctors did not always trust the accuracy of anemia tests. The health personnel asserted that most doctors were well trained in the administration of medical management, but had limited nutrition knowledge regarding therapeutic diets and treatment options, thus, reducing referral to the dietitian. Additionally, most facilities did not have early detection protocols and services as part of postnatal care. Hospitals at the district and subdistrict levels lacked iron/anemia‐specific programs but instead focused on broad nutrition and health issues. This was partly because healthy personnel considered anemia a symptom of an infectious ailment and not as a disease. Hospitals and clinics, especially in rural settings, reported a lack of access to iron and folate supplements for patients with severe anemia. Most hospitals in Ghana did not have resident dietitians who could supervise therapeutic diet treatments for anemia. The district hospitals reported that due to high caseloads, they were unable to frequently conduct household visits and outreach in their surrounding communities. The personnel cited that their facility was only able to provide outreach services once a year.

## Discussion

Addressing all basic, underlying, and immediate causes of anemia in the conceptual framework of malnutrition is essential to decreasing the prevalence of anemia and improving the overall health of children in Ghana. Countries in Asia, Latin America, and the Caribbean have made progress to combat some of these causes of anemia and malnutrition.^20^ Program examples from these continents can inform the current programs being implemented in Ghana, as well as address the missing gaps in implementation and prevention. All organizations implemented projects addressing at least one underlying cause of anemia. The contextual basic causes of malnutrition from UNICEF's conceptual framework^2^ indirectly affected anemia prevention and treatment; however, few programs reported insight on how their programs affected child health. Ghana has a stable economy; however, commitment and prioritization of nutrition issues that cause anemia is lacking.

From this assessment, the researchers deduced gaps in anemia treatment and prevention to include inadequate human resources, inadequate waste management, and sanitation services, and insufficient funding for implementation of projects (Fig. 1). UNICEF's conceptual framework shows that multiple stakeholders should be part of the prevention and treatment of malnutrition in Ghana.^2^ However, overlaps in the type of interventions being implemented within the same communities were observed indicating a clear lack of integration and communication between the programs. Several organizations were simultaneously implementing broad nutrition education in the same communities while neglecting complementary, but equally important programs addressing anemia prevention. Suboptimal coordination did not result in the judicious use of program funding and resources in the implementation of anemia prevention and treatment strategies, requiring multisectoral approach of programming.

As part of food‐based interventions, fruit intake is important for anemia prevention, because of the role of vitamin C in iron absorption. VC is an antioxidant and its consumption in mixed meals enhances the bioavailability of nonheme iron.^9^ In low‐income populations, nonheme iron from plant‐based diets is common. From this assessment, programs provided pregnant women with folic acid and iron supplements to improve iron status according to the WHO standard practice. Supplementation through micronutrient tablets and MNPs to young children in Kenya,^21^ Nepal,^14^ and pregnant and lactating women in India^22^ have shown the potential to improve anemia among children in the short term. However, supplementation programs in areas where cereals and teas are commonly consumed need to stress the importance of antinutritive factors on the bioavailability of iron. In these areas, providing iron and plant sources without VC supplements or promotion of fruit consumption may negate the benefit of iron supplements provided. Children raised in vegan and vegetarian households may have unique challenges in maintaining optimum iron status.^9^, ^23^ However, very little information is available about the percentage of households with these dietary practices in Ghana. A smaller cross‐sectional study (*n* = 26) conducted among older Ghanaian children (9–11 years) who consumed vegetarian diets showed unique gaps in vitamin B_12_ but not iron intake,^24^ thus, requiring more research.

Most food processors used staple cereal and grains in their commercial food products. However, these food products likely contain antinutritive factors or may contain low levels of iron. Heme iron from animal sources is more bioavailable;^9^ however, neither of the food manufacturers who were interviewed included animal source products. Studies in Bangladesh explored the use of fish^25^ and goat meat powders in Malawi.^26^ Milk is not a rich source of iron, although it may be one of the most commonly consumed animal source foods in the area. The *Obaasima* project identified in this study is part of the multicountry ANF4W project implemented in Ghana, Kenya, Tanzania, and Bangladesh. The ANF4W project has the potential to improve the MN status of women and children.^27^, ^28^ Additionally, lipid‐based nutrient supplements and *plumpy’* nuts contain milk, peanuts, and vegetable oils, although GHS did not routinely distribute these supplements to children.

The FDA is a governmental agency that regulates nutrition food labels as well as fortification of food across all regions of Ghana, thus, helping to improve food safety and inadequate dietary intake. The FDA evaluates the nutrition labeling of processed packaged foods to ensure that they meet requirements and fortification guidelines. The FDA also requires mandatory fortification of wheat flour with MNs, including iron, thus, encouraging a higher intake of nutrients within the population of Ghana and preventing malnourishment.^29^ The FDA and GSA regulate infant formula formulation and labeling, as well as supplements. Limitation of these agencies in the policing of labeling was reported in the case of iodine‐related products in Ghana,^30^ thus, a similar gap is foreseen for iron‐related products. Additionally, low consumption of fortified foods is reported for rural households.^31^

Inadequate health personnel to provide health services was another reported challenge. Trained staff were frequently transferred to new locations interrupting the benefits of on‐the‐job training and the continuity of care. According to the GHS facts and figures report in 2017, only 102 nutrition officers were engaged across the 10 regions of Ghana.^32^ Inadequate personnel, and/or untrained about anemia‐related issues professionals is a critical setback in reducing the prevalence of childhood anemia in Ghana. This gap translates to insufficient personnel to contribute to program design, development, and implementation. Most clinics in rural areas are run by community health workers. The task burden of community health workers in Ghana and the rest of Sub‐Saharan Africa is extremely high^33^ because they are expected to provide all first aid and treatment services at the community level. The SRING project, a completed multicountry project implemented in 13 countries focused on addressing the human resource gap in Ghana from 2012 to 2018.^19^ Additionally, the GHS has provided CHPS compounds in deprived communities with Community Health Works (CHWs) and incentives to address the issue of limited human resources. To address similar human resource issues, the Thailand Health Department turned to training local community members to spread essential health knowledge and carry out preventive efforts “with a focus on maternal and child health and nutrition.” Surveillance of anemia levels in Thailand indicated over a 15% decrease in anemia prevalence among school‐age children throughout the time of this implementation, which was done in addition to the distribution of iron supplements.^34^ The CHPS compounds in Ghana provide similar services as volunteers in Thailand. Thus, this concept is worth exploring in Ghana, in addition to the Ghana government's prioritization of health personnel training. Similarly, more collaboration between universities that trained health personnel and government through the GHS could curb this gap. From the personnel interviewed, about 75% were trained nutritionists/dietitians. This is encouraging, however, there are benefits to include personnel of diverse background, such as policy experts, agriculturists, and environmental scientists, in anemia programs because of the multifaceted causes of anemia.

Ghanaian children were consistently exposed to malaria, worm infection,^35^ and other risk factors, thus, early warning strategies should be incorporated in surveillance systems managed by the GHS. For example, regular testing of children could be incorporated as a part of growth monitoring and promotion programs. In this assessment, we observed that regular testing to serve as an early warning of anemia was not conducted. To facilitate the early warning, equipment could be made available and acclimatized in the field setting. The RING project reported that they addressed this gap by distributing hemocues and on‐the‐job training on how to use and interpret the results in field/rural settings. However, the commonly used hemocue only provides measurements of hemoglobin, a nonspecific biomarker of iron status.^9^ Similarly, no routine screening exists for nonnutrition causes of anemia, such as thalassemia, sickle cell anemia, cancer, and other diseases.^9^ These conditions, however, could be contributing to the high levels of anemia recorded in Ghana.

Current governmental efforts to improve access to clean water and overall sanitation and hygiene are needed, as well as waste management. Insanitary conditions increase the risk of infections, making individuals more susceptible to becoming undernourished and anemic.^13^ Studies in Kenya confirm gaps in WASH policies, and translation to communities, especially in health facilities,^13^, ^36^ that serve as models for households. In Ghana, efforts by the district assemblies to enforce compliance with the GSA guidelines need to be intensified to ensure access to sanitation and water services in homes, hospitals, social venues, and at work. These initiatives at the district level, with a simultaneous strengthening of the community capacity and systems,^19^ and the government's financial commitment^20^ would contribute to sustainable reduction in anemia rates.

## Conclusions and recommendations

In comparison with the immediate and underlying causes of malnutrition, very few projects addressed the reported basic causes, namely inadequate human resources, housing/water/toilet facilities (*n* = 3 projects), and poverty/financial resources (*n* = 2 projects). Multiple programs, sometimes with an overlapping focus in the same region, were addressing anemia. The main gaps identified by researchers were insufficient communication between project implementers. In the design phase, new projects need to prioritize communication with existing projects to promote efficient use of limited funding and human resources. New programs may focus on improving financial investment in programs and communities since few related programs currently exist. Projects focused on training new and existing health personnel are urgently needed to match the increasing needs of communities. Access to good road networks in rural farming communities will enhance the free flow of agricultural food produce and improve access to food and advanced health care during emergencies.

Furthermore, because several programs in Ghana targeted the improvement of food security through the promotion of homestead and dry season gardening, repurposing programs to encompass both education about the local MN‐rich foods and the processing and preparation skills required to prepare healthy meals would aid in decreasing MN deficiencies. An earlier study in Ghana on irrigation projects shows multiple benefits for improving malnutrition and food insecurity.^11^ In Nepal, homestead food production that included the distribution of MNPs improved anemia rates among children.^14^ Investments by government and donors on irrigation and homestead projects may be sustainable solutions to the lean season of fresh foods in Northern Ghana. Instead of general nutritional education to populations, specific and contextualized messaging on available, low‐cost food sources in the context of poverty, seasonality, pica practices, gender and cultural practices, and food insecurity would be critical.^31^, ^35^, ^37^ VC intake from fruits should be highlighted, especially in rural communities.^31^ Small‐ and medium‐scale food manufacturers should explore affordable animal‐source commercial food products.

## Limitations

Different data collection (in‐person, phone, and email) approaches may have affected the depth of data shared. A method was used based on the availability of staff, thus, allowing the opportunity to interview more programs during data collection. The limitations with email responses were that extensive probing could not be done in the responses. However, only one respondent used the method to provide valuable information for this assessment.

Owing to the limitations in time and participant availability, the research team was unable to interview other organizations that could contribute broader insight and show indirect links to childhood anemia. Some of these institutions include Ghana Water and Sanitation, the Departments of Food and Agriculture, Industry, Social Welfare, Environmental Protection, and the Roads and Highways Authority.

## Authors’ contributions

B.A.Z.A. developed the study concept. K.A.P., A.S., and B.A.Z.A. finalized the study design and coordinated participant recruitments. R.S., O.G., N.B., and B.A.Z.A. collected, cleaned, and analyzed the data. B.A.Z.A. drafted the manuscript. K.A.P., A.S., R.S., O.G., and N.B. reviewed and provided inputs to the manuscript.

## Competing interests

K.A.P. works at the University of Cape Coast, Ghana, and A.S. works with PointHope. Research participants were recruited in both organizations. However, participating organizations did not receive or provide any benefits for their contributions. The remaining authors declare no competing interests.

## Supporting information


**Supplementary File S1**. Compiled Interview Guides Administered To Key Informants.

## References

[1] Stevens, G.A. , M.M. Finucane , L.M. De‐Regil , *et al*. 2013. Global, regional, and national trends in haemoglobin concentration and prevalence of total and severe anaemia in children and pregnant and non‐pregnant women for 1995–2011: a systematic analysis of population‐representative data. Lancet Glob. Health 1: 316–325. 10.1016/S2214-109X(13)70001-9 PMC4547326 25103581

[2] United Nations Children's Fund (UNICEF) . 1992. UNICEF Annual Report 1992. Accessed September 9, 2018. https://www.unicef.org/about/history/files/unicef_annual_report_1992.pdf.

[3] Ghana Statistical Service (GSS) , Ghana Health Service (GHS), and ICF International . 2015. Ghana Demographic and Health Survey 2014. Rockville, MD: GSS, GHS, and ICF International.

[4] University of Ghana, GroundWork, University of Wisconsin‐Madison, KEMRI‐Wellcome Trust, UNICEF . 2017. Ghana Micronutrient Survey 2017. Accra, Ghana. Accessed September 12, 2020. https://www.unicef.org/ghana/media/1276/file/UN368291.pdf.

[5] Smith, J.L. & S. Brooker . 2010. Impact of hookworm infection and deworming on anaemia in non‐pregnant populations: a systematic review. Trop. Med. Int. Health 15: 776–795. 20500563 10.1111/j.1365-3156.2010.02542.x PMC2916221

[6] Wegmüller, R. , H. Bentil , J.P. Wirth , *et al*. 2020. Anemia, micronutrient deficiencies, malaria, hemoglobinopathies and malnutrition in young children and non‐pregnant women in Ghana: findings from a national survey. PLoS One 15: e0228258. 31999737 10.1371/journal.pone.0228258 PMC6991996

[7] Bird, J.K. , R.A. Murphy , E.D. Ciappio , *et al*. 2017. Risk of deficiency in multiple concurrent micronutrients in children and adults in the United States. Nutrients 9. 10.3390/nu9070655. PMC5537775 28672791

[8] Ntenda, P.A. , O. Nkoka , P. Bass , *et al*. 2018. Maternal anemia is a potential risk factor for anemia in children aged 6–59 months in Southern Africa: a multilevel analysis. BMC Public Health 18: 650. 29788935 10.1186/s12889-018-5568-5 PMC5964691

[9] Gibson, R.S. 2005. Principles of Nutritional Assessment. New York: Oxford University Press.

[10] Talukder, A. , K.A. Osei , J.N. Haselow , *et al*. 2014. Contribution of homestead food production to improved household food security and nutrition status – lessons learned from Bangladesh, Cambodia, Nepal and the Philippines. In VI Improving Diets and Nutrition: Food‐Based Approaches. B. Thompson & L. Amoroso , Eds.: 58–73. Rome: CAB International and FAO.

[11] Steiner‐Asiedu, M. , B.A.Z. Abu , J. Setoglo , *et al*. 2012. The impact of irrigation on the nutritional status of children (0–59 mo) in the Sissala West District of the Upper West Region of Ghana. Curr. Res. J. Soc. Sci. 4: 86–92.

[12] Kothari, M.T. , A. Coile , A. Huestis , *et al*. 2019. Exploring associations between water, sanitation, and anemia through 47 nationally representative demographic and health surveys. Ann. N.Y. Acad. Sci. 1450: 249–267. 31232465 10.1111/nyas.14109 PMC6771505

[13] Ngure, F.M. , M.B. Reid , J.H. Humphrey , *et al*. 2014. Water, sanitation, and hygiene (WASH), environmental enteropathy, nutrition, and early child development: making the links. Ann. N.Y. Acad. Sci. 1308: 118–128. 24571214 10.1111/nyas.12330

[14] Osei, A.K. , P. Pandey , D. Spiro , *et al*. 2015. Adding multiple micronutrient powders to a homestead food production programme yields marginally significant benefit on anaemia reduction among young children in Nepal. Matern. Child Nutr. 11(Suppl. 4): 188–202. 25682798 10.1111/mcn.12173 PMC6860240

[15] Rivera, J.A. , T. Shamah , S. Villalpando , *et al*. 2010. Effectiveness of a large‐scale iron‐fortified milk distribution program on anemia and iron deficiency in low‐income young children in Mexico. Am. J. Clin. Nutr. 91: 431–439. 20016011 10.3945/ajcn.2009.28104

[16] Altucher, K. , M.K. Rasmussen , M.E. Bardern , *et al*. 2005. Predictors of improvement in hemoglobin concentration among toddlers enrolled in the Massachusetts WIC Program. J. Am. Diet Assoc. 105: 709–715. 15883545 10.1016/j.jada.2005.02.010

[17] Brito, A. , M. Olivares , T. Pizarro , *et al*. 2013. Chilean complementary feeding program reduces anemia and improves iron status in children aged 11 to 18 months. Food Nutr. Bull. 34: 378–385. 24605687 10.1177/156482651303400402

[18] SPRING . 2017. Understanding anemia: guidance for conducting a landscape analysis. 2nd ed. Arlington, VA: Strengthening Partnerships, Results, and Innovations in Nutrition Globally (SPRING) project.

[19] SPRING . 2017. Reducing anemia in Ghana: the SPRING approach and lessons learned. Accessed September 12, 2020. https://www.spring-nutrition.org/sites/default/files/

[20] Development Initiatives . 2018. 2018 Global Nutrition Report: shining a light to spur action on nutrition. Bristol, UK: Development Initiatives.

[21] Leyvraz, M. , D.M. David‐Kigaru , C. Macharia‐Mutie , *et al*. 2018. Coverage and consumption of micronutrient powders, fortified staples, and iodized salt among children aged 6 to 23 months in selected neighborhoods of Nairobi County, Kenya. Food Nutr. Bull. 39: 107–115. 29284306 10.1177/0379572117737678

[22] Sachdev, H.P. & T. Gera . 2013. Preventing childhood anemia in India: iron supplementation and beyond. Eur. J. Clin. Nutr. 67: 475–480. 23388662 10.1038/ejcn.2012.212

[23] Pawlak, R. & K. Bell . 2017. Iron status of vegetarian children: a review of literature. Ann. Nutr. Metab. 70: 88–99. 28319940 10.1159/000466706

[24] Osei‐Boadi, K. , A. Lartey , G.S. Marquis & E.K. Colecraft . 2012. Dietary intakes and iron status of vegetarian and nonvegetarian children in selected communities in Accra and Cape Coast, Ghana. Afr. J. Food Agr. Nutr. Dev. 12: 5822–5842.

[25] Campbell, R.K. , K.M. Hurley , A.A. Shamim , *et al*. 2016. Effect of complementary food supplementation on breastfeeding and home diet in rural Bangladeshi children. Am. J. Clin. Nutr. 104: 1450–1458. 27680994 10.3945/ajcn.116.135509 PMC5081719

[26] Theophilus, R.J. , M. Miller , W.H. Oldewage‐Theron & J. Dawson . 2019. The Winning Weaning Food (WWF): the development of a complementary food for food‐insecure infants and young children in Malawi. Nutrients 11. 10.3390/nu11102292. PMC6835519 31557966

[27] Namdiero, A. & N. Martin . 2015. Affordable Nutritious Foods for Women (ANF4W): overview figure 1: ANF4W partner countries innovative approaches for alleviating micronutrient deficiencies in women of childbearing age. Sight and Life Field report. Accessed October 17, 2020. https://sightandlife.org/wp-content/uploads/2015/11/SAL_Mag_Frontiers_In_Nutrition_2015_ANF4W_overview.pdf.

[28] Gavin‐Smith, B. & D. Amankwah . 2018. A demand driven approach to reduce micronutrient malnutrition in Ghana. Sight and Life. Accessed October 19, 2020. https://sightandlife.org/wp-content/uploads/2018/12/35_SALMZ_0218_FieldReport_03.pdf.

[29] Nyumuah, R.O. , T.C. Hoang , E.F. Amoaful , *et al*. 2012. Implementing large‐scale food fortification in Ghana: lessons learned. Food Nutr. Bull. 33(4 Suppl.): S293–S300. 23444710 10.1177/15648265120334S305

[30] Abu, B. , W. Oldewage‐Theron & R. Aryeetey . 2019. Risks of excess iodine intake in Ghana: current situation, challenges, and lessons for the future. Ann. N.Y. Acad. Sci. 1446: 117–138. 30489642 10.1111/nyas.13988 PMC6618322

[31] Abu, B.A.Z. , J.E. Raubenheimer & V. van den Berg Louise . 2020. Iron‐focussed nutritional status of mothers with children (6-59 months) in rural northern Ghana. Heliyon. 4;6(6):e04017. 10.1016/j.heliyon.2020.e04017. 32529061 PMC7283160

[32] Ghana Health Service (GHS) . 2017. Ghana Health Service – 2016 Annual Report. Accessed September 9, 2018. https://www.ghanahealthservice.org/downloads/GHS_ANNUAL_REPORT_2016_n.pdf.

[33] Philips, M. , R. Zachariah & S. Venis . 2008. Task shifting for antiretroviral treatment delivery in Sub‐Saharan Africa: not a panacea. Lancet 371: 682–684. 18295026 10.1016/S0140-6736(08)60307-4

[34] Winichagoon, P . 2002. Prevention and control of anemia: Thailand experiences. J. Nutr. 132: 862S–866S. 11925499 10.1093/jn/132.4.862S

[35] Abu, B.A.Z. , L.V. van den Berg , J.E. Raubenheimer , *et al*. 2017. Pica practices among apparently healthy women and their young children in Ghana. Physiol. Behav. 177: 297–304. 28442334 10.1016/j.physbeh.2017.04.012

[36] Abu, T.Z. & S.J. Elliott . 2020. When it is not measured, how then will it be planned for? WaSH a critical indicator for universal health coverage in Kenya. Int. J. Environ. Res. Public Health 17. 10.3390/ijerph17165746. PMC7460032 32784498

[37] Abu, B.A.Z. , V.J. Louw , A. Dannhauser , *et al*. 2013. Knowledge attitudes and practices (KAP) regarding iron deficiency (ID) among mothers in an anemia endemic population in Northern region of Ghana. Poster Presentations. Matern. Child Nutr. 9(Suppl. 3): 42–55.

