# Supplementary material for: Qualitative assessments of anemia‐related programs in Ghana reveal gaps and implementation challenges
Source: Ann N Y Acad Sci. 2020 Dec 24;1492(1):27–41. doi: 10.1111/nyas.14538 (PMC8246908; doi:10.1111/nyas.14538)
Supplement: Supplementary file 1 — Supplementary File S1. Compiled Interview Guides Administered To Key Informants. [file NYAS-1492-27-s001.docx]

**Compiled Interview Guides Administered To Key Informants.**

| **Sociodemographic** | Name of organisation…………………….………..…………………..…………  Name of respondent…………………………….…………………………………  Respondent’s position in the organization…….………………………………….  Highest education obtained…………………………………………..  Gender: Male/ Female  Email/Phone contact……………………..………………………………………… |
| --- | --- |
| **KEY INFORMANT GROUPS** | **QUESTIONS** |
| **Implementing agencies and projects** | 1. How is your organisation linked with childhood anemia prevention/treatment/management? 2. How long has your organisation been involved with childhood anemia prevention? 3. Childhood anemia-related interventions/programs:   For each program implemented, probe:   - - 1. What is the scope of the programs you deliver?     2. Program type?     3. Probe on education on iron content/excess intake in meals?     4. Regions in Ghana covered?     5. What is the source of funding?   **Immediate causes**   - Describe the role inadequate dietary intake in childhood anemia prevention. List the programs your organisation have addressing these issues? - Describe the role of diseases in childhood anemia status? List the programs your organisation have addressing these issues?   **Underlying causes**   - Discuss the role of food security in anemia prevention? List the programs your organisation have addressing these issues? - Discuss the roles of inadequate food safety, feeding and care practices? List the programs your organisation have addressing these issues? - Discuss the role of housing, environment and health services? Do your organisation have any programs addressing these?   **Basic causes**   - Describe the role of inadequate access to services childhood anemia prevention? List the programs your organisation have addressing these issues? - Describe the role of inadequate financial resources in childhood anemia prevention/treatment? List the programs your organisation have addressing these issues? - Describe the role of inadequate human resources in childhood anemia prevention/treatment? List the programs your organisation have addressing these issues? - Describe the role of social cultural setting/practices in childhood anemia prevention/treatment? List the programs your organisation have addressing these issues? - Describe the role of economic and political content in childhood anemia prevention/treatment? List the programs your organisation have addressing these issues?  1. Describe the short term consequence of childhood anemia in the communities you work in? List the programs your organisation have addressing these issues? 2. Describe the long term consequence of childhood anemia in the communities you work in? List the programs your organisation have addressing these issues? 3. What in your experience are the major challenges in the childhood anemia prevention? 4. What are the gaps and challenges of anemia prevention implementation? 5. What other projects/intervention activities are likely to contribute to iron intake? 6. In your experience, what surveillance system exists to monitor iron intake, particularly anemia and iron deficiency anemia? 7. Have you received any training on the following: a) Iron content in meals? b) Bioavailability of iron? c) Iron intake? 8. If yes, who gave the training? What was the content of training? 9. What lessons can be learned from the current childhood anemia prevention/treatment programs your organisation run? 10. **Request for a copy of reports and materials, if they exists** |
| Regulatory agencies | QUESTIONS: Please response to the questions that are applicable to you.  How is your organisation linked with childhood anemia prevention/treatment/management?  How long has your organisation been involved with childhood anemia prevention or treatment?  Childhood anemia-related interventions/programs:  What is the scope of the programs you deliver?  Program type? Probe for sanitation and food safety, iron, folate and vitamin C, supplements and iron in food.  Probe on education on iron content/excess intake in meals?  Regions in Ghana covered?  What is the source of funding?  Describe the standards/regulatory guidelines the organisation use to monitor iron and vitamin C in foods?  How are these standards/guidelines implemented?  Describe the long term consequence of childhood anemia in the communities you work in? List the programs your organisation currently implement that are addressing these issues long term consequences of anemia?  What in your experience are the major challenges in the implementation of the regulatory guidelines?  What in your experience are the major challenges in childhood anemia prevention in general?  What do you think are the gaps in the standard/regulation or its implementations regarding these nutrients?  How do you suggest these gaps be addressed?  **Request for a copy of reports/nutrient profiles including iron, if they exists** |
| **Medical facilities and biomedical Laboratories** | 1. In your experience, what tests are being used to diagnose suspected anemia cases in your facility?    1. Probe: childhood anemia 2. Which of the following cases have you observed or tested in the past 5 years?    1. Iron deficiency anemia    2. Anemia   **(request for a copy of records/reports of cases, if they exists)**   1. Are these tests/diagnosis done routinely? 2. **Childhood anemia related interventions/programs:**   For each program implemented, probe:   1. What is the scope of the programs you deliver? 2. Program type? 3. Probe on education on iron content/excess intake in meals? 4. Regions in Ghana covered? 5. What is the source of funding?   **Immediate causes**   - Describe the role inadequate dietary intake in childhood anemia prevention. List the programs addressing these issues? - Describe the role of diseases in childhood anemia status? List the programs addressing these issues?  1. Describe the short term consequence of childhood anemia in the communities you work in? List the programs your organisation have addressing these issues? 2. Describe the long term consequence of childhood anemia in the communities you work in? List the programs your organisation have addressing these issues? 3. What in your experience are the major challenges in the childhood anemia prevention? 4. What do you think are the gaps and challenges of anemia prevention implementation? 5. Kindly provide reference to any other **units/departments** in your facility that could provide useful information on risk anemia in children from the past 5 years. 6. Are there any other procedures in your department where iron status is monitored? What are these, please give details? 7. Is there any counselling done before or during anemia diagnosis?   Probe for information given to patients or their families on iron status or iron in diet?   1. What are the types of follow-ups/monitoring done with patients whose iron status have been monitored? Probe for referral to the diet therapy units of the hospital. 2. **Request for a copy of reports/nutrient profiles including iron, if they exists.** |
| **Food producers (small, large, and prepared food companies)** | 1. What foods do you produce at ____________________________ 2. Which of these foods does your organisation add iron or vitamin C to? 3. How does your organisation monitor the amount of iron in the food you produce? 4. Have you received any training for the workers / distributors regarding iron content in in foods/iron intake? 5. If yes, who gave the training and what was the content of the training? 6. Describe the short term consequence of childhood anemia in the communities you work in? List the programs your organisation have addressing these issues? 7. Describe the long term consequence of childhood anemia in the communities you work in? List the programs your organisation currently have that are addressing these issues? 8. What in your experience are the major challenges in the childhood anemia prevention? 9. What do you think are the gaps and challenges of anemia prevention implementation? 10. **Request for a copy of reports/nutrient profiles including iron, if they exists.** |
